# Notes from the Field: Severe Human Metapneumovirus Infections — North Dakota, 2016

**DOI:** 10.15585/mmwr.mm6618a7

**Published:** 2017-05-12

**Authors:** Claire M. Midgley, Jill K. Baber, Holly M. Biggs, Twila Singh, Michelle Feist, Tracy K. Miller, Kirby Kruger, Susan I. Gerber, John T. Watson, Molly A. Howell

**Affiliations:** ^1^Division of Viral Diseases, National Center for Immunization and Respiratory Diseases, CDC; ^2^Epidemic Intelligence Service, CDC; ^3^North Dakota Department of Health, Division of Disease Control, Bismarck, North Dakota.

On May 27, 2016, CDC was informed by North Dakota Department of Health of a recent cluster of severe respiratory illnesses that included two deaths in children at a large hospital (hospital A) in Fargo, North Dakota, caused by human metapneumovirus (HMPV). An investigation was initiated to explore possible risk factors for illness. HMPV is a cause of both upper and lower respiratory tract infections, including bronchiolitis and pneumonia, particularly among young children (*1*) and older adults (*2*). In the United States, the typical HMPV season extends from November–February through May–July (*3*). No vaccine is currently available to prevent HMPV infection.

Six HMPV-positive pediatric inpatients (median age = 2.5 years) were identified at hospital A during April–May 2016. Diagnostic tests were performed at a commercial laboratory using a reverse transcription–polymerase chain reaction (RT-PCR)–based respiratory virus panel (RVP). The number of HMPV infections detected and the percentage of HMPV-positive respiratory virus panels from hospital A did not appear high compared with the same period in 2015 (hospital A, unpublished data, 2015 and 2016). Among the six patients identified in 2016 (Table), five had underlying medical conditions, including premature birth (three), congenital heart disease (three), bronchopulmonary dysplasia (two), developmental delay (three), and cerebral palsy (two). Four children required mechanical ventilation, and two of the four had acute respiratory distress syndrome and pneumothorax. Two of the six patients died; both had considerable medical comorbidities. Four of the patients were American Indian; all four survived, although two required mechanical ventilation and two required supplemental oxygen. Two of the four American Indian children were transferred to hospital A from an Indian Health Service facility. During preliminary discussions with the North Dakota Department of Health, local Indian Health Service personnel did not describe a notable increase in respiratory illness during the investigation period, although testing for HMPV was not routinely done.

**TABLE:** Selected demographic and clinical characteristics of pediatric (aged <18 years) and adult inpatients with laboratory-confirmed human metapneumovirus infection — six hospitals, North Dakota, July 31, 2015–May 26, 2016

Characteristic, median (range)	Pediatric cluster, hospital A	Other pediatric cases	Adult cases
	(N = 6)	(N = 11)	(N = 27)
Age group	2.5 yrs (4 mos–9 yrs)	10 mos (2 mos–9 yrs)	69 yrs (49–95 yrs)
Length of hospitalization (days)	8.5 (2–47)	3 (1–11)	5 (1–38)

**Characteristic, no. (%)**			
Male sex	2 (33)	7 (64)	9 (33)
Reside in long-term care facility	0 (0)	0 (0)	10 (37)
Ever smoker	0 (0)	0 (0)	22 (81)
**Race**			
White	2 (33)	10 (91)	27 (100)
American Indian	4 (67)	0 (0)	0 (0)
Unknown	0 (0)	1 (9)	0 (0)
Underlying medical conditions reported*			
None	1 (17)	2 (18)	0 (0)
Chronic lung disease^†^	2 (33)	7 (64)	19 (70)
Chronic heart disease^§^	0 (0)	0 (0)	16 (59)
Congenital heart disease	3 (50)	1 (9)	0 (0)
Immunocompromised^¶^	1 (17)	0 (0)	5 (19)
Premature birth	3 (50)	4 (36)	0 (0)
Developmental delay	3 (50)	2 (18)	0 (0)
Genetic condition	2 (33)	1 (9)	1 (4)
Cerebral palsy	2 (33)	0 (0)	0 (0)
Diabetes	0 (0)	0 (0)	7 (26)
Chronic kidney disease	0 (0)	0 (0)	5 (19)
Hemodialysis	0 (0)	0 (0)	2 (7)
**Common signs/Symptoms**			
Cough	4 (67)	11 (100)	23 (85)
Fever (reported)	4 (67)	8 (73)	19 (70)
Stuffy nose/Congestion	1 (17)	10 (91)	3 (11)
Wheezing	5 (83)	2 (18)	10 (37)
Shortness of breath/Rapid or shallow breathing	3 (50)	3 (27)	21 (78)
Vomiting/Nausea	3 (50)	4 (36)	2 (7)
**Clinical findings at admission**			
Fever at admission (>100.4°F [>38.0°C])	2 (33)	4 (36)	3 (11)
Tachycardia (physician reported)	1 (17)	3 (27)	7 (26)
Tachypnea (physician reported)	1 (17)	4 (36)	6 (22)
Abnormal breathing sounds	4 (67)	6 (55)	20 (74)
Crackles	2 (33)	2 (18)	4 (15)
Wheezes	3 (50)	5 (45)	18 (67)
**Co-detected viruses**			
Coronavirus	0 (0)	1 (9)	0 (0)
Respiratory syncytial virus	0 (0)	1 (9)	0 (0)
Rhinovirus or enterovirus	1 (17)	2 (18)	1 (4)
**Maximum respiratory support required**			
Mechanical ventilation	4 (33)	1 (9)	2 (7)
Noninvasive ventilation**	0 (0)	0 (0)	8 (30)
Supplemental oxygen	2 (33)	8 (73)	13 (48)
No oxygen support	0 (0)	2 (18)	4 (15)
**Medication**			
Bronchodilator	6 (100)	7 (64)	25 (93)
Steroid	4 (67)	6 (55)	19 (70)
Antiviral	0 (0)	0 (0)	1 (4)
Antibiotic	6 (100)	6 (55)	26 (96)
**Outcome**			
Died	2 (33)	0 (0)	3 (11)

* Some patients had multiple underlying conditions.

^†^ Chronic lung disease included asthma, reactive airway disease, bronchopulmonary dysplasia, chronic obstructive pulmonary disease, or emphysema, or the requirement for home oxygen combined with other lung conditions such as chronic respiratory failure or pulmonary hypertension.

^§^ Chronic heart disease included congestive heart failure, diastolic heart failure, coronary artery disease, aortic stenosis, and arrhythmias. Reports of isolated hypertension were not included.

^¶^ Immunocompromised patients included those with an immune deficiency, such as hypogammaglobulinemia, or those taking immunosuppressive medications.

** Includes continuous positive airway pressure or bilevel positive airway pressure.

Case finding was expanded to five additional large hospitals throughout North Dakota. A case was defined as a positive HMPV test in any pediatric or adult inpatient since June 1, 2015. In addition to the six cases initially reported, 11 pediatric cases from three hospitals and 27 adult patients from four hospitals were identified (Table). Medical chart abstractions were performed.

Among the 11 additional pediatric patients (median age = 10 months), none were American Indian. Nine had underlying medical conditions, including chronic lung disease (seven) and premature birth (four). One patient required mechanical ventilation; none died.

Among the 27 adult patients (median age = 69 years), all were white, and all had underlying medical conditions, particularly chronic lung disease (19) or chronic heart disease (16). This finding is consistent with previous descriptions of HMPV infection in hospitalized adults, in which elderly patients and those with underlying medical conditions had a more complicated clinical course (*4*). Twenty-two patients were current or previous smokers. Ten patients required either mechanical ventilation (two) or noninvasive ventilation (eight); among these 10 patients, nine reported chronic lung disease. Three adult patients died. Although 10 patients resided in long-term care facilities before hospital admission, no HMPV clusters were identified.

HMPV can cause severe respiratory illness in children and adults. Increased HMPV diagnostic testing could facilitate enhanced understanding of the clinical spectrum of illness, virus circulation, and populations at increased risk. Four of the six children in the hospital A cluster were American Indian. Although American Indian children are at increased risk for hospitalization with respiratory syncytial virus (*5*), whether HMPV disproportionately affects this population is unknown. Further study is needed to understand the epidemiology of HMPV in the American Indian population.
